# Flexible age-period-cohort modelling illustrated using obesity prevalence data

**DOI:** 10.1186/s12874-020-0904-8

**Published:** 2020-01-28

**Authors:** Annette Dobson, Richard Hockey, Hsiu-Wen Chan, Gita Mishra

**Affiliations:** The University of Queensland, School of Public Health, Brisbane, Queensland Australia

**Keywords:** Age-period-cohort effects, Statistical modelling, Australian women, Obesity

## Abstract

**Background:**

Use of generalized linear models with continuous, non-linear functions for age, period and cohort makes it possible to estimate these effects so they are interpretable, reliable and easily displayed graphically. To demonstrate the methods we use data on the prevalence of obesity among Australian women from two independent data sources obtained using different study designs.

**Methods:**

We used data from two long-running nationally representative studies: seven cross-sectional Australian National Health Surveys conducted between 1995 and 2017–18, each involving 6000–8000 women; and the Australian Longitudinal Study on Women’s Health which started in 1996 and involves more than 57,000 women in four age cohorts who are re-surveyed at three-yearly intervals or annually. Age-period-cohort analysis was conducted using generalized linear models with splines to describe non-linear continuous effects.

**Results:**

When analysed in the same way both data sets showed similar patterns. Prevalence of obesity increased with age until late middle age and then declined; increased only slightly across surveys; but increased steadily with birth year until the 1960s and then accelerated.

**Conclusions:**

The methods illustrated here make the estimation and visualisation of age, period and cohort effects accessible and interpretable. Regardless of how the data are collected (from repeated cross-sectional surveys or longitudinal cohort studies), it is clear that younger generations of Australian women are becoming heavier at younger ages. Analyses of trends in obesity should include cohort, in addition to age and period, effects in order to focus preventive strategies appropriately.

## Background

In many public health contexts, it is important to be able to distinguish between age, period, and cohort (APC) effects as drivers of temporal changes. For example, the introduction of new laws or taxes to reduce tobacco smoking would be expected to affect smokers in different age groups and different generations at the same time, that is, to produce period effects. In contrast, adoption of wearable devices to monitor physical activity might occur first in younger people, that is, a cohort or generational effect.

There is ample evidence that the prevalence of overweight and obesity is increasing globally [1] and there are growing concerns about the consequences for the health of populations and health care costs in the future. Yet there is conflicting evidence about whether period or cohort effects predominate. There is evidence that younger cohorts are heavier than previous generations in high income countries such as the United States [2], United Kingdom [3], and Australia [4] as well as some middle income countries such as China [5]. In Europe, however, trends in obesity-attributable mortality exhibit cohort effects in some countries but not others [6].

The distinctions between APC effects in overweight and obesity are important for two reasons. Firstly, from the perspective of prevention, measures that may affect energy intake across a population (such as changes to the food supply, through industry regulation or taxation, or interventions such as dietary guidelines and food labelling) might be expected to produce period effects. In contrast, if younger people are affected differently from older people, due to lifestyle differences or social pressures, cohort differences will be apparent and health promotion activities can be targeted accordingly. The second reason why it is important to understand APC effects is to forecast future levels of overweight and obesity and consequential demands on health services. If all cohorts are assumed to behave similarly over time and they do not, the predictions will be misleading.

Despite the global increase in body mass index, commentators in some countries have interpreted the available data as suggesting that the growth of the population prevalence of overweight and obesity across age groups has slowed or even that prevalence has stabilized [7, 8]. These differences of opinion may be due, in part, to how the data are collected and summarized. For example, if data from repeated cross-sectional surveys are compared by age and sex, then generational (or birth cohort) differences may not be apparent. If the same data were re-arranged in cohort format, generational differences may be easier to identify. If longitudinal data are available from people with a wide age range followed over a long time, it may be possible to distinguish period effects from cohort differences.

A challenge in estimating the magnitude of APC effects is the identifiability problem that occurs because the year of birth, which defines the generation or cohort, is linearly related to age at any given observation time or period, as follows: cohort = period – age. To manage this problem various constraints are typically used in the analysis, but different constraints can lead to different results [9]. More recently, hierarchical generalized linear mixed models have been advocated to manage the identifiability problem [10], but there remains controversy about their ability to accurately distinguish APC effects [11]. Additionally, most published studies of APC effects in overweight and obesity have used categorical or a combination of categorical and continuous variables with a variety of constraints. Use of numerous categorical variables can be problematic because the large number of parameters to be estimated results in wide confidence intervals and lack of reliability. Consequently, the models may produce inconsistent results, for example between trends in overweight and obesity [12]. However, if the effects are non-linear then second order effects are identifiable, though constraints are still needed to obtain interpretable estimates of the whole effect [13]. An approach to APC modelling that involves treating all three variables as continuous and using restricted cubic splines to model non-linear patterns, has considerable advantages. Estimates for the three components can be interpreted individually (with suitable constraints on estimates for the other components) and they can be combined to give predicted rates; each of the components (adjusted for the others) can be depicted graphically; modelling can be extended by the addition of covariates; and the approach can be broadened to projection of future rates [14, 15].

In this paper we illustrate the analysis of APC effects using data on the prevalence of obesity among women in Australia. The prevalence of obesity can reasonably be assumed to be driven by gradual changes in the population, including lifestyles and food supplies. This makes use of continuous APC terms particularly appropriate. We use two data sets from large nationally representative samples analysed separately: repeated cross-sectional data from the Australian National Health Surveys since 1995 [16, 17]; and longitudinal data from four cohorts of participants in the Australian Longitudinal Study on Women’s Health that commenced in 1996 [18, 19]. Firstly, we present four plots commonly used in cancer epidemiology to explore APC effects [20]. Secondly, we fit generalized linear models with continuous variables for each of the APC effects. Finally, using the models, we show graphs summarising each of the effects and discuss how these should be interpreted.

## Methods

### Data sets

The Australian Bureau of Statistics (ABS) has conducted seven National Health Surveys (NHS) since 1995 [16, 17]. These are nation-wide cross-sectional surveys each with 6000–8000 female participants aged 18 years and over. The surveys conducted in 1995, 2007–08, 2011–12, 2014–15 and 2017–18 included measured heights and weights, whereas those in 2001 and 2004–05 had only self-reported heights and weights.

The Australian Longitudinal Study on Women’s Health (ALSWH) began in 1996 with the recruitment of more than 47,000 women in three age groups: women aged 18–23 years (born 1973–73, *n* = 14,247), 45–50 years (born 1946–51, *n* = 13,714) and 70–75 years (born 1921–26, *n* = 12,432). These women were randomly sampled from the database of the Australian universal health insurance scheme, now called Medicare Australia, which includes all residents of Australia. Since then, they have been surveyed on average every 3 years, initially by mailed questionnaires and more recently with the option of online completion of the surveys. Details of the study methods and representativeness of the samples have been published elsewhere [18]. In 2013, another cohort of women then aged 18–23 (born 1989–95, *n* = 17,012) was recruited using a variety of methods and these women have been surveyed annually using a web-based questionnaire [19]. At every survey, women are asked to report their weight and height. Women who were pregnant at the time of completing the survey were asked to report their pre-pregnancy weight (except for the first three surveys of the 1973–78 cohort where pregnant women’s weight was treated as missing). Height and weight data were collected over the following periods: 1989–95 cohort, 2013–2017; 1973–78 cohort, 1996–2018; 1946–51 cohort, 1996–2016; 1921–26 cohort, 1996–2011 (because these items were not asked for these elderly women after that date. Response and attrition rates for each survey are available at the study website http://www.alswh.org.au/.

### Measures

From both data sources, body mass index (BMI) was calculated as weight (kilograms) divided by the square of height (metres). The World Health Organization classification was used, namely: underweight BMI < 18.5 kg/m^2^, normal weight BMI 18.5–24.99 kg/m^2^, overweight BMI 25–29.99 kg/m^2^, and obese BMI ≥ 30 kg/m^2^. In this study, we focus on obesity because other studies have shown that excess burden on the healthcare system is largely associated with obesity rather than overweight [21].

### Statistical methods

The prevalence of obesity for each year of age was calculated for each ALSWH cohort using information provided by participants at each survey (i.e., each period). This means that most ALSWH participants contributed data at multiple periods. Prevalence of obesity from the NHS was extracted from age-group and sex specific data in various ABS publications and summary tables [16, 17]. The NHS data were re-arranged using a Lexis diagram [20] to create synthetic cohorts centred at ages comparable with the ALSWH cohort surveys.

For each data set the prevalence of obesity was then presented in four plots:

- Prevalence by age for different periods;
- Prevalence by period for different age groups;
- Prevalence by age for different cohorts;
- Prevalence by cohort for different age groups.

If plots a) and b) both show parallel curves this supports an age-period model and if plots c) and d) show parallel curves this supports an age-cohort model [20].

Based on evidence from these plots, APC models were fitted using the Stata procedure *apcfit* described by Rutherford, Lambert and Thompson [14]. This method uses a generalized linear model framework with age, period, and cohort treated as continuous variables. The number of obese people was modelled using a Poisson distribution with a log link function (to give rate ratios), an offset given by log(number of people surveyed), and functions for age, period, and cohort as the explanatory variables. This model is based on the assumption that the observations are independent. This is reasonable for the NHS data which were from new samples at each survey. But the ALSWH participants contributed repeated observations, so the samples at each age and period/cohort time are not independent; this could lead to bias, particularly underestimation of variability.

To obtain functions for age, period and cohort restricted cubic splines are used with transformations to the spline basis vectors for period and cohort terms [20]. Due to the systematic difference between the NHS self-reported data on height and weight in the 2001 and 2004–5 surveys and the measured data from the other surveys, we omitted the former from the modelling.

For each data set models were fitted with terms for: age, period and cohort; age and period; and age and cohort. Period effects were estimated relative to the reference year of 2007, the median year for the ALSWH survey data, and cohort effects were estimated relative to 1951, the median year of birth for ALSWH participants. Due to the identifiability problem for first order effects APC models are over-parameterised, and for the type of models considered here three constraints are needed [20]. The choice of constraints does not affect the model fit but does affect the estimates and hence the graphical displays of effects. If, as for obesity, age is a major unmodifiable factor, the age function is of primary importance. A linear temporal change, or drift, can be arbitrarily attributed to either the cohort function or the period function. The age function can be represented in two ways:

APC: As age-specific rates for a particular period, after adjustment for cohort effects;

ACP: As age-specific rates for a particular cohort, after adjustment for the period effect.

For the APC version the drift is included in the period function. The period function is set to zero for the reference date and the period effects are relative risks relative to that date. The cohort function has both the average and the slope set to zero and represents a residual relative risk relative to fitted values for age and period effects. This model shows the cross-sectional age-specific rates at the reference year and how the pattern varies over time.

For ACP version the drift is included in the cohort function. The cohort function is set to zero at the reference birth year and the cohort effects are relative risks relative to that year. The period function has both the average and the slope set to zero and represents a residual relative risk relative to fitted values for age and cohort effects. The model can be interpreted as showing the biological or longitudinal effect of age for the reference cohort and how this differs across cohorts.

Model fit was assessed using the Akaike Information Criterion (AIC) and Bayesian Information Criterion (BIC) – smaller absolute values indicate better fit. The log-likelihood values and degrees of freedom (d.f.) were also used to calculate the deviance = (− 2 × the difference in log-likelihood values for the nested models) and obtain *p*-values using the chi-squared distribution. To assess period and cohort effects the fit of models without each of these terms (i.e., models with A + C and A + P) was compared with the fit of the model with A + P + C. The numbers of knots for the cubic splines were selected using AIC, BIC and the following principles: parsimony (using as few parameters as needed to capture the main features but avoid overfitting); the same number of equally spaced internal knots for cohort and period effects so these are treated symmetrically; and the same number of nodes for both data set, in order to facilitate comparisons.

## Results

### Prevalence estimates

**Fig. 1:**
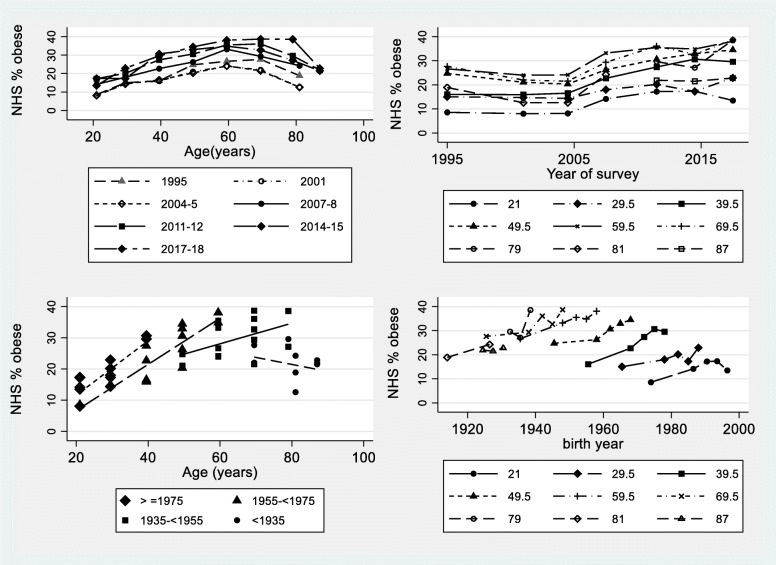
Prevalence of obesity among women who participated in the Australian National Health Surveys (NHS) conducted in 1995 (measured heights and weights), 2001 (self-reported heights and weights), 2004–5 (self-reported), 2007–8 (measured), 2011–12 (measured), 2014–15 (measured) and 2017–18 (measured); approximately 6000–8000 women at each survey. **a** (top left) by age for each survey; **b** (top right) by survey for selected ages; **c** (bottom left) by age for synthetic cohorts defined by year of birth and plotted by quartiles of year of birth; **d** (bottom right) by cohort for selected ages and excluding self-reported data from the 2001 and 2004–5 surveys

Prevalence of obesity among women obtained from successive National Health Surveys is shown in Fig. 1. Panel a) (top left) is the usual form of presentation, namely by age for each survey. Prevalence increased with age and then declined with the peak shifting to older ages in more recent surveys. It is clear that the self-reported data tend to systematically underestimate obesity making data from the 2001 and 2004–5 surveys not comparable with the other surveys, so these data were not included in the APC modelling. However, the curves in both panels a) and b) (top right) are somewhat parallel, providing some evidence of period effects. Similarly panels c) (bottom left) and d) (bottom right) are suggestive of parallel effects for younger cohorts, but not for older cohorts, suggesting non-linear cohort effects.

**Fig. 2:**
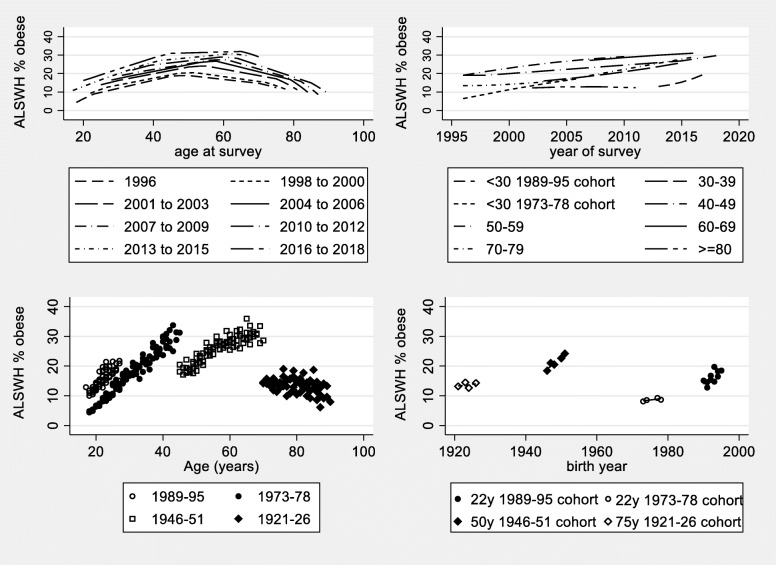
Prevalence of obesity among participants in the Australian Longitudinal Study on Women’s Health (ALSWH) born in 1989–95, 1973–78, 1946–51 and 1921–26 and surveyed approximately every 3 years (self-reported heights and weights). **a** (top left) by age for survey waves shown as smoothed curves (fractional polynomials); **b** (top right) by year of survey by age group shown as smoothed curves (fractional polynomials); **c** (bottom left) by age for the four cohorts defined by year of birth; **d** (bottom right) by cohort for selected ages (with the largest numbers of observations)

Prevalence of obesity among ALSWH participants is shown in Fig. 2. Panel c) (bottom left) is the usual form of presentation for longitudinal data. The four cohorts show distinctively different patterns. The percentage of obese women in the cohort born in 1989–95 was much higher when they were aged 18–23 than in the 1973–78 cohort when they were the same age. The rates of increase in obesity were greater for women in the two younger cohorts than for women born in 1946–51, and prevalence in the oldest cohort actually declined. These distinctions are not clear when the data are presented by age for different surveys as in panel a) (top left). Nevertheless panel a) does show the non-linear age effect clearly and the curves are approximately parallel, increasing with successive surveys. Similarly the curves in panel b) (top right) show increases with year of survey, suggestive of period effects. Panel d) (bottom right) illustrates a limitation of these data, namely that the time span of surveys is far less than the time span of years of birth.

In summary, the exploratory analyses show very similar patterns for the two data sets when the data are displayed in the same way. In both data sets there are strong non-linear age effects and some evidence of both cohort and period effects, both also potentially non-linear. To investigate these effects further, APC models were fitted.

### Age-period-cohort models

To describe the non-linear age effect five equally spaced internal knots were selected. For the period and cohort effects four knots were needed, again reflecting non-linearity. The fit of the models is shown in Table 1. For the smaller NHS data set evidence for either a period or a cohort effect, is weak and not statistically significant, whereas for the ALSWH data there is strong evidence of both effects. For both data sets support for a cohort effect is more pronounced than for a period effect.

**Table 1:** Fit statistics for age–period–cohort models

	AIC	BIC	Log-likelihood	d.f.	Deviance	p-value
National Health Survey						
A + P + C	9.185	− 40.237	− 161.516	25		
A + C	9.185	−45.158	− 164.512	28	5.992	0.112
A + P	9.236	−43.229	− 165.476	28	7.920	0.048
Australian Longitudinal Study on Women’s Health						
A + P + C	7.275	− 1565.563	− 1194.653	319		
A + C	7.352	− 1551.528	− 1210.378	322	31.450	< 0.001
A + P	7.429	− 1525.734	− 1223.276	322	57.246	< 0.001

Akaike Information Criterion (AIC), smaller values indicate better fit; Bayesian Information Criterion (BIC), smaller absolute values indicate better fit; log-likelihood (smaller absolute values indicate better fit); d.f. is degrees of freedom; deviance = − 2× (difference in log-likelihood from A + P + C model) approximately has a chi-squared distribution with d.f. = (difference in d.f. from the A + P + C model) = 3 in all cases here; *p*-value for deviance.

**Fig. 3:**
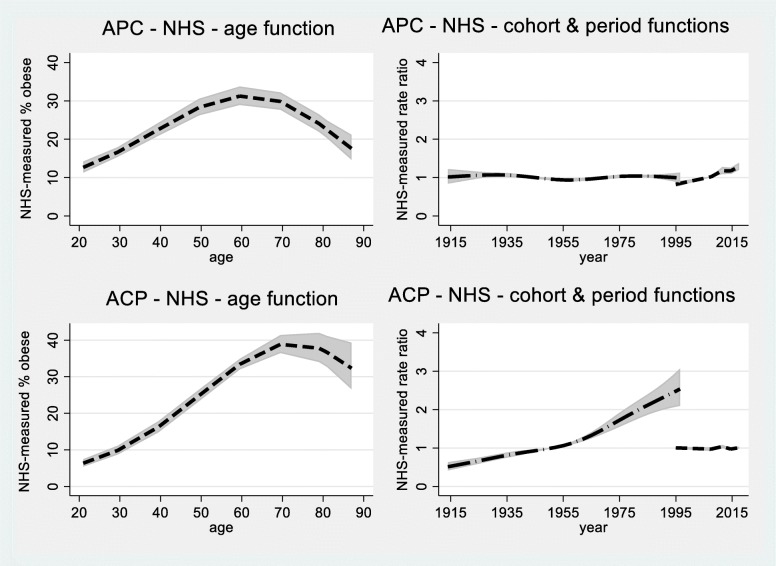
Estimates of age, cohort and period effects from NHS data (omitting 2001 and 2004–5), fitted values and 95% confidence intervals, from models with age, period and cohort effects. **a** (top left) estimated age-specific rates for the reference year of 2007; **b** (top right) the function on the right is the period function relative to 2007 (including drift) and the function on the left is a cohort function representing residual effects; **c** (bottom left) estimated age-specific rates for women born in 1951; **d** (bottom right) the function on the left is the cohort function (including drift) relative to women born in 1951 and the function on the right is a period function representing residual effects

**Fig. 4:**
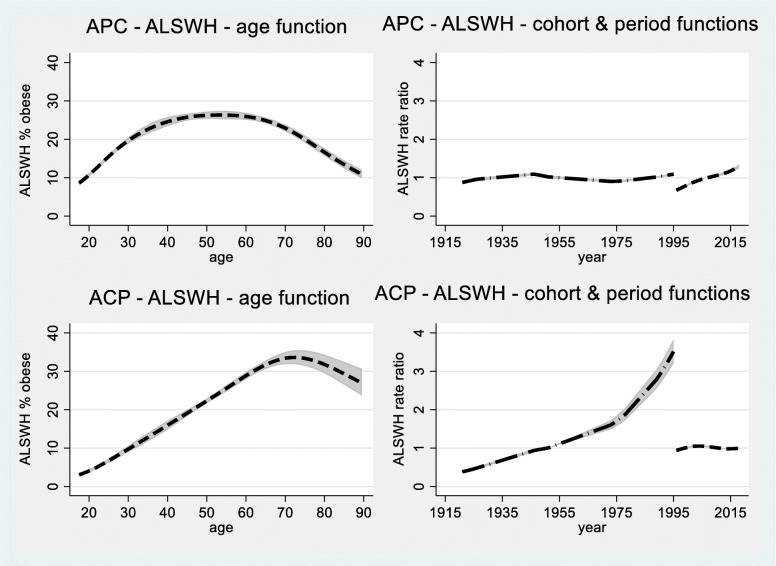
Estimates of age, cohort and period effects from ALSWH data, fitted values and 95% confidence intervals, from models with age, period and cohort effects. **a** (top left) estimated age-specific rates for the reference year of 2007; **b** (top right) the function on the right is the period function relative to 2007 (including drift) and the function on the left is a cohort function representing residual effects; **c** (bottom left) estimated age-specific rates for women born in 1951; **d** (bottom right) the function on the left is the cohort function (including drift) relative to women born in 1951 and the function on the right is a period function representing residual effects

The fitted values from the models with age, period and cohort effects are shown in Fig. 3 for the NHS data and Fig. 4 for the ALSWH data. Estimates from the NHS data are less smooth and the confidence intervals are wider due to fewer observations. Overall, the patterns in Figs. 3 and 4 are very similar. As expected from Table 1, cohort functions (from ACP models) show more pronounced trends than period functions (from APC models).

In both Figures panels a) (top left) and b) (top right) show the results relative to the reference year of 2007 obtained from an APC model, and panels c) (bottom left) and d) (bottom right) show results relative to the cohort born in 1951 obtained from the ACP model. Figure 3 panel a) shows the age pattern that was apparent in the exploratory plots in Fig. 1, with a gradual increase until about age 60 and then a decline. Figure 3 panel b) shows that even when the drift is included in the period function (the graph on the right) the function only increased slightly over time (corresponding to Fig. 1 b) if the 2001 and 2004–5 data were omitted). Figure 3 panel c) shows that for women born in 1951 obesity prevalence increased markedly with age until about 70 and then declined. The graph on the left in Fig. 3 panel d) shows that the cohort effect (including drift) increased steadily until the 1960s and then accelerated.

In Fig. 4 the same patterns are apparent. For both Figures comparing panels a) and c) shows how the usual cross-sectional plot (panel a) from APC models) masks cohort differences (panel b) from ACP models) and this may be of practical importance.

## Discussion

Using data from multiple cross-sectional national surveys and a national longitudinal study of Australian women, we have shown substantially the same patterns in the prevalence of obesity. Obesity increases with age until late mid-age and then decreases. Successive generations are increasingly obese. There is less evidence of a period effect, although this should be interpreted cautiously as the time span of the surveys (about 20 years) is much shorter than the time span for years of birth (about 70 years).

One particular contribution of this paper is to illustrate a method for estimating APC effects, when they can reasonably be modelled by continuous non-linear variables, using explicit assumptions to deal with the non-identifiability problem. Another is to depict the results of modelling in a manner that facilitates interpretation. The method can be extended to include covariates such as sex and ethnicity and interaction terms [14], and it can be used for projections [15]. Compared to other approaches, there are fewer parameters to estimate, changes are modelled as smooth functions of time, and the same number of parameters can be used for period and cohort effects so they can be directly compared. Additionally, the method is suitable for relatively sparse data. From the perspective of obesity research, the value of this approach is greatly enhanced by the graphical output that summarises the main effects.

Consistency between results obtained by creating synthetic cohort data from multiple cross-sectional survey data and longitudinal cohort data has been found before for the US and UK [22, 23]. Similarly, the age pattern we identified is consistent with previous findings of increasing BMI with age up to about 70 years followed by a decline [24]. Furthermore, the importance of cohort differences has been reported in the United States [2], United Kingdom [3] and Australia [25].

The higher prevalence of obesity in NHS data compared to the ALSWH data is to be expected. Firstly, the ALSWH data are self-reported and, as can be seen from the NHS data for the 2001 and 2004–5 surveys, prevalence of obesity calculated from self-reported data tends to be lower than from measured data. A recent report by the ABS compared measured and self-reported values for heights and weights and showed the extent of underestimation of obesity from calculated BMI: 21.5% vs. 26.8% for women in 2017–18, with the difference increasing with age of participants but not having changed much since 1995 [17]. Other studies have found that self-reported weight is underestimated by 0.2–3.4 kg and that the overweight and obese people tend to underestimate weight more than healthy weight people [26, 27]. In addition, there was some over-representation of university educated women in ALSWH at survey 1 and this bias had increased at survey 6 [28]. We have shown that higher educational attainment is associated with lower initial weight and BMI, and less weight gain over time [29].

Finally, we have provided evidence of generational differences in obesity among Australian women. This makes future predictions especially uncertain, as the contributions from future generations is unknown, and population changes are complex due to immigration, declining fertility and population aging. From a health promotion perspective, however, the challenge is to halt the escalation of BMI in successive generations.

## Conclusion

The methods used to model age, period and cohort effects in this paper can provide valuable insights into the obesity epidemic. Provided they are presented in the same way (using Lexis diagrams) population data from repeated cross-sectional surveys or longitudinal cohort studies can produce consistent results. Using continuous variables to describe temporal patterns in the prevalence of obesity, it is clear that the phenomenon varies with age, across time (period effect) and across generations (cohort effect). For example, Australian women born in 1989–95 had higher prevalence of obesity than women born in 1973–78 when they were the same age. The implication of these findings is that preventive strategies should take account of generational differences (as well as socioeconomic and other factors).

## Data Availability

The data sets generated and use during this current study are available in the Mendeley Data repository, doi:10.17632/tkrcdcj44v.1

## Abbreviations

ABS: Australian Bureau of Statistics
ACP: Age-cohort-period
AIC: Akaike Information Criterion
ALSWH: Australian Longitudinal Study on Women’s Health
APC: Age-period-cohort
BIC: Bayesian Information Criterion
BMI: Body mass index
d.f: Degrees of freedom
NCD: Non-communicable diseases
NHS: (Australian) National Health Survey
